# Taurine Biosynthesis in a Fish Liver Cell Line (ZFL) Adapted to a Serum-Free Medium

**DOI:** 10.3390/md15060147

**Published:** 2017-05-25

**Authors:** Chieh-Lun Liu, Aaron M. Watson, Allen R. Place, Rosemary Jagus

**Affiliations:** 1 Institute of Marine and Environmental Technology, University of Maryland Center for Environmental Science, 701 E. Pratt Street, Baltimore, MD 21202, USA; liuh@umces.edu; 2 Marine Resources Research Institute, South Carolina Department of Natural Resources, 217 Fort Johnson Rd, Charleston, SC 29412, USA; WatsonA@dnr.sc.gov

**Keywords:** taurine-free medium, ZFL cells, taurine biosynthesis

## Abstract

Although taurine has been shown to play multiple important physiological roles in teleosts, little is known about the molecular mechanisms underlying dietary requirements. Cell lines can provide useful tools for deciphering biosynthetic pathways and their regulation. However, culture media and sera contain variable taurine levels. To provide a useful cell line for the investigation of taurine homeostasis, an adult zebrafish liver cell line (ZFL) has been adapted to a taurine-free medium by gradual accommodation to a commercially available synthetic medium, UltraMEM™-ITES. Here we show that ZFL cells are able to synthesize taurine and be maintained in medium without taurine. This has allowed for the investigation of the effects of taurine supplementation on cell growth, cellular amino acid pools, as well as the expression of the taurine biosynthetic pathway and taurine transporter genes in a defined fish cell type. After taurine supplementation, cellular taurine levels increase but hypotaurine levels stay constant, suggesting little suppression of taurine biosynthesis. Cellular methionine levels do not change after taurine addition, consistent with maintenance of taurine biosynthesis. The addition of taurine to cells grown in taurine-free medium has little effect on transcript levels of the biosynthetic pathway genes for cysteine dioxygenase (CDO), cysteine sulfinate decarboxylase (CSAD), or cysteamine dioxygenase (ADO). In contrast, supplementation with taurine causes a 30% reduction in transcript levels of the taurine transporter, TauT. This experimental approach can be tailored for the development of cell lines from aquaculture species for the elucidation of their taurine biosynthetic capacity.

## 1. Introduction

Taurine (2-amino ethanesulfonic acid) is an abundant β-amino acid found in a variety of vertebrate tissues that is essential for cardiovascular function and vision [1]. In mammals, the physiological functions of taurine include the formation of carboxylic conjugates of bile and a variety of xenobiotics [2,3], anti-inflammatory and anti-apoptotic activity [4,5,6], antioxidant defense [7], osmoregulation [8,9,10], membrane stabilization [11,12], and neuronal development [13,14,15].

Taurine is considered semi-essential in most species [4] and only essential in strict carnivores, such as cats [16,17]. Species lacking sufficient endogenous synthesis require dietary supplementation. As in mammals, taurine plays important roles in many physiological processes in fish including bile salt conjugation [18], osmoregulation [19], and vision [20,21]. In addition, dietary taurine supplementation has been shown to stimulate growth in multiple fish species, such as rainbow trout [22]; Japanese flounder [18,23]; turbot [24]; red seabream [25,26]; cobia [27,28,29,30,31]; Atlantic salmon [32]; and yellowtail [33].

**Figure 1:**
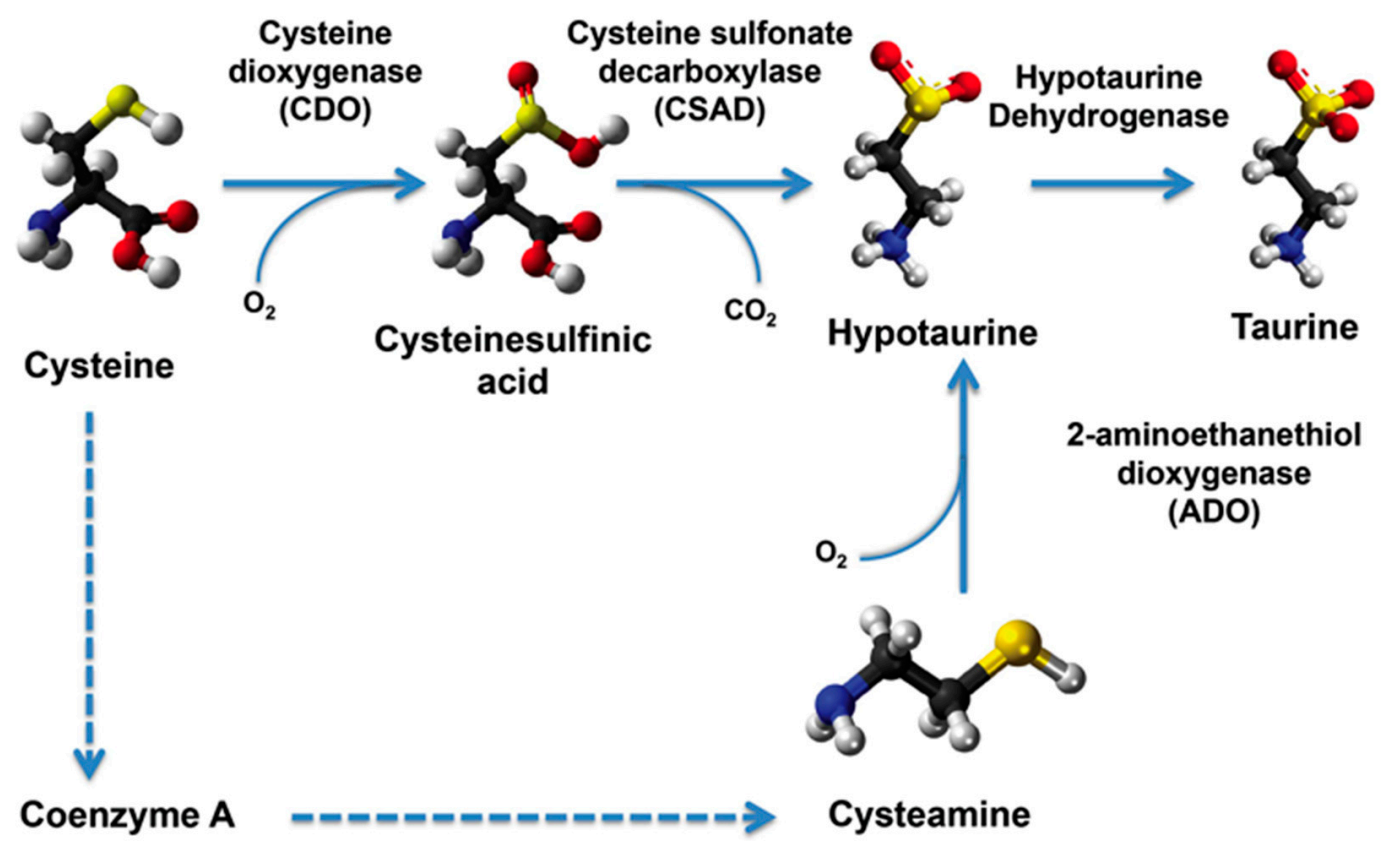
Taurine biosynthetic pathway from methionine-derived cysteine.

In contrast to animals, taurine is found only at very low levels in plants—less than 1% of the levels found in animal tissues [34]. Because of the need to replace fishmeal in aquafeeds with plant ingredients, taurine requirements in fish diets have been investigated in multiple aquaculture species, as has the taurine biosynthetic pathway (reviewed in [35]). In vertebrates, the major pathway of taurine biosynthesis is from sulfur-containing amino acids such as methionine and cysteine (Figure 1) (reviewed in [35]). In animals, methionine-derived homocysteine is used as a sulfur source and its condensation product with serine (cystathionine) is converted to cysteine. The major pathway of taurine biosynthesis involves the sequential oxidation of cysteine to cysteine sulfinic acid by cysteine dioxygenase (CDO), followed by decarboxylation by cysteine sulfonate decarboxylase (CSAD), and oxidation of the resulting hypotaurine to taurine. However, other possible pathways have been proposed, including production of cysteic acid from cysteine sulfinic acid without hypotaurine production and subsequent decarboxylation by a presumptive cysteic acid decarboxylase (CAD) to form taurine [35]. In addition, hypotaurine could be produced via oxidation of cysteamine, the end product of coenzyme A degradation, via oxidation by 2-aminoethanethiol dioxygenase (ADO) [35].

In addition to the enzymes responsible for synthesis, a highly conserved membrane transporter is critical in the transport and recycling of taurine [36]. In general, tissue taurine levels are regulated by the circulating plasma taurine levels and an increase or decrease in renal TauT expression and activity [36]. TauT activity is upregulated when circulating taurine levels are low, to increase the reabsorption and recycling of existing taurine, and downregulated when plasma taurine levels are high to allow maintenance of appropriate concentrations. Animals capable of regulating TauT expression and activity do so rapidly after dietary taurine intake or changes in dietary protein or taurine content [37].

Cell lines provide an important biological tool for carrying out investigations into areas such as physiology, virology, toxicology, carcinogenesis, but also can be useful for deciphering biosynthetic pathways and their regulation. However, their usefulness in conducting investigations into taurine homeostasis is compromised by the fact that current cell culture media all contain taurine provided either by fetal serum (FBS) and/or as a component of the medium (reviewed in [38,39]). In order to test whether taurine levels influence fish cell growth and expression of genes involved in taurine synthesis, we needed to use a taurine-free, and therefore serum-free, medium to grow the cells. To achieve this goal, we have successfully adapted an adult zebrafish liver cell line (ZFL) to serum-free synthetic medium (UltraMEM™-ITES), allowing us to control taurine levels. Unlike many carnivorous fish, the omnivorous zebrafish has a complete taurine biosynthetic pathway and is not dependent on taurine in the diet, being able to convert methionine via cysteine into taurine [40]. The usefulness of ZFL cells adapted to serum-free medium was demonstrated by an investigation of the effect of taurine on growth rate, expression of the genes involved in taurine biosynthesis and transport, as well as the relationship between taurine and methionine levels.

## 2. Results

### 2.1. Taurine Concentrations in L-15 Medium and Fetal Bovine Serum (FBS)

Despite the fact that taurine is not listed as a component of Leibovitz’s L-15, the taurine level is 11 ± 0.8 μM (Table 1). In addition, the taurine level in FBS (Atlanta Biologicals, Lawrenceville, GA, USA) is 20 ± 4 μM, giving an overall 12 μM in L-15/ supplemented with 9% FBS. In order to provide taurine-free conditions, we sought an alternate taurine-free medium from which serum was excluded. A commercially available serum-free medium, UltraMEM™-ITES (Lonza, Walkersville, MD, USA) was assessed for suitability. Taurine is undetectable in this medium (Table 1), potentially providing conditions under which the effects of exogenous taurine could be tested directly.

### 2.2. Adaptation of ZFL Cells to Growth in Serum-Free Medium

Supplementing basal culture media with animal serum of different origins is essential for cell growth, metabolism, and to stimulate proliferation [38,39]. To address cell requirements in serum-free conditions, insulin, transferrin, ethanolamine, and selenium supplements (ITES) have been developed (reviewed in [39]). Insulin functions as a growth factor that helps cells utilize glucose and amino acids [39]. Transferrin is a universal iron carrier that provides iron and also helps cells maintain iron homeostasis [42]. Ethanolamine is required for phospholipid synthesis and selenium is required for proper functioning of glutathione peroxidase, thioredoxin reductase, and other antioxidant enzymes [43,44]. Even with ITES supplementation, cultured cells need to undergo a gradual adaptation process that involves progressive adaptation to lower serum concentrations until serum-free conditions are reached [39]. This gradual accommodation is necessary because abrupt withdrawal of serum induces a swift response across different cell types by compromising mTOR and other signal transduction pathways, reducing protein synthesis and halting cell growth (reviewed in [45]).

**Figure 2:**
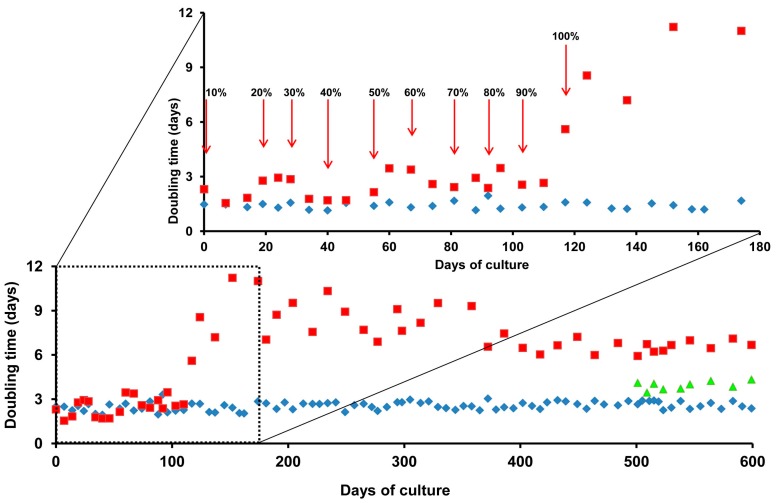
Doubling times of adult zebrafish liver cell line (ZFL) cells during adaptation to growth in UltraMEM™-ITES. ZFL cells were exchanged into decreasing percentages of L15-FBS and increasing percentages of UltraMEM™-ITES. Medium substitutions were made every second passage. Cell counts were taken every 2–8 days. Doubling times were calculated for ZFL growing in L15-FBS alone (blue diamonds), in L15-FBS supplemented with a range of UltraMEM™-ITES concentrations (red squares), or in UltraMEM™-ITES, HEPES-KOH supplemented with 2 mM taurine (green triangles).

The adaptation of ZFL cells to growth in 100% UltraMEM™-ITES was assessed by monitoring cell doubling time during the adaptation period, as shown in Figure 2. The exchange from L15-FBS to 90% L-15-FBS/10% UltraMEM™-ITES had no effect on ZFL cell doubling time. With each successive exchange from 20 to 90% UltraMEM™-ITES, cell doubling time increased approximately two-fold, with the cells adapting to the new medium formulation over 7–10 days and returning to the initial doubling time of 2.54 ± 0.28 days. After 123 days of adaptation, ZFL cells were switched from 90 to 100% UltraMEM™-ITES. This exchange resulted in an increased doubling time to 12 days. However, over the next 250 days and with the inclusion of the commonly used culture medium buffering agent, *N*-2-hydroxyethylpiperazine-*N*-2-ethane sulfonic acid (HEPES-KOH, 20 mM, pH 7), the cells adjusted and adopted a stable doubling time of 6.6 ± 0.43 days, approximately 2.5 times that of the initial doubling time in L15-FBS. After 500 days of adaptation, doubling time could be decreased to 3.9 ± 0.26 days after further supplementation with 2 mM taurine or increased methionine.

### 2.3. Effect of Taurine Supplementation on Amino Acid Pools in Cells and Medium

ZFL cells, fully adapted to growth in 100% UltraMEM™-ITES, were incubated with UltraMEM™-ITES containing 0, 12 μM, 160 μM, and 2 mM taurine for 24 h. A comparison of amino acid concentrations in the medium before and after incubation with ZFL cells for 24 h is shown in Table 2. We chose 12 μM as representative of the taurine level found in L-15/FBS; 160 μM as representative of the level found in zebrafish [41]; 2 mM as representative of a typical taurine concentration used to examine taurine effects in cultured cells (supplementation with 0.1–20 mM reported). In general, there was little change in the media concentrations of amino acids after 24 h with or without taurine supplementation. The exceptions are the rapid and dramatic depletion of methionine, as well as the elevated level of alanine from excretion by the cells. The increase in taurine reflects that added to supplement the medium.

The cellular levels of amino acids (normalized to recovered norleucine) are shown in Table 3. Because the average volume of ZFL cells is not known, amino acid levels are expressed as pmol per 3 × 10^7^ cells. Even in the absence of taurine supplementation, the intracellular taurine level was 315 ± 63 pmol per 3 × 10^7^ cells, indicating a robust ability to synthesize taurine. Supplementation of the medium with 12 μM taurine, the concentration found in L15/FBS, increased intracellular taurine 1.8-fold (Table 4).

Increasing taurine concentration in the medium to 160 μM or 2 mM increased taurine levels 2.8- and 3.2-fold, respectively, approaching saturation. Our expectation was that this would be reflected in decreased hypotaurine levels reflecting suppression of cellular taurine biosynthesis. Surprisingly, at any level of taurine supplementation, intracellular hypotaurine actually increased 1.5-fold with increasing media taurine, remaining reasonable constant at any concentration of taurine supplementation, suggesting little suppression of cellular taurine biosynthesis. Our expectation was that supplementation with taurine would spare methionine and potentially lead to increases in intracellular methionine. In fact, at any level of taurine supplementation, intracellular methionine decreased by approximately 10%. There appeared to be no effect on other cellular amino acid pools with taurine supplementation except perhaps on arginine, although this may be a measurement artifact of the large taurine levels masking arginine quantification. It should be noted that the amino acid analysis method used converts glutamine and asparagine to their respective acids so values presented represent both amino acid forms.

### 2.4. Transcript Levels of Taurine Transporter and Taurine Biosynthesis Pathway Genes

To examine whether added taurine affected transcript abundance of the genes involved in taurine biosynthesis, cells were treated without or with 160 μM taurine. After 24 h, the cells were collected and RNA extracted for quantitative reverse transcription PCR (RT-qPCR) analysis of taurine transporter (TauT), cysteine dioxygenase (CDO), cysteine sulfinic acid (CSA), and cysteine sulfonate decarboxylase (CSAD) transcripts.

**Figure 3:**
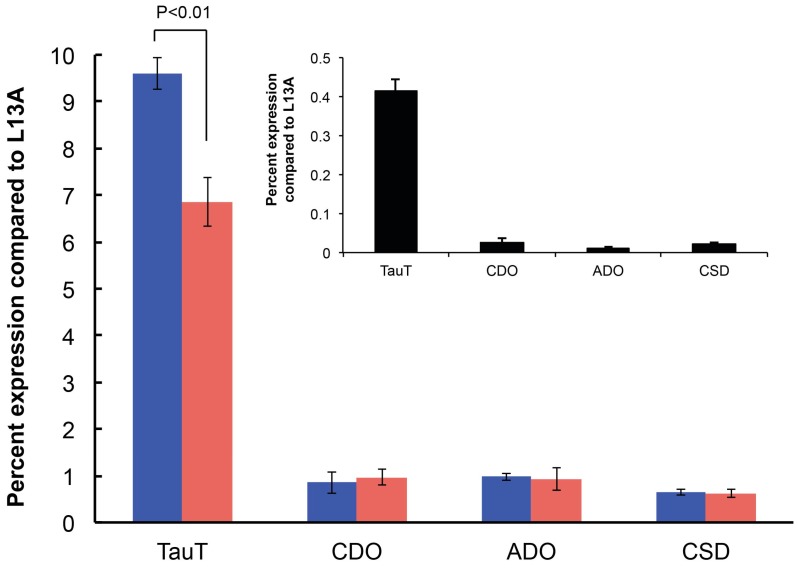
Transcript levels of taurine pathway and taurine transporter genes in ZFL cells growing in L15/FBS versus UltraMEM™-ITES (±taurine). Quantitative RT-PCR was performed using cDNA from 10 ng RNA and primers given in Table 2. qPCR was performed using a 7500 Real Time PCR System (Applied Biosystems, Foster City, CA, USA) with SYBR green fluorescent label. Reactions included Taqman™ Universal master mix (Bio-Rad, Hercules, CA, USA), 1:100 SYBR green (100 U stock), 5 μM of each primer. Expression levels of zebrafish cysteamine dioxygenase (ADO), cysteine dioxygenase (CDO), cysteine sulfinate decarboxylase (CSAD), taurine transporter protein (TauT) are expressed relative to 60S ribosomal protein L13A transcript levels. Data are presented as the mean ± S.D. (*n* = 3 replicates).

The transcript levels were expressed relative to those of ribosomal protein L13A (Figure 3). The relative expression of TauT, ADO, CDO, and CSAD to the reference transcript was largely influenced by the lower level of L13A transcript abundance in cells growing in UltraMEM™-ITES compared to those growing in L15-FBS (Figure 3, inset). The addition of taurine to the taurine-free medium had little effect on the expression of the biosynthetic pathway genes, ADO, CDO, and CSAD. However, a 30% reduction was seen in transcript levels of the taurine transporter, TauT, presumably to decrease the uptake of taurine into the cells. Such a response has also been reported in rat astrocytes in which the taurine content in cultured cells varies greatly according to the concentration of taurine in the extracellular medium [46]. As in rat astrocytes, taurine supplementation downregulates TauT transcript levels in ZFL cells but not those for taurine biosynthetic enzymes.

### 2.5. Growth Effects of Taurine Are Dependent on Methionine Concentration

**Figure 4:**
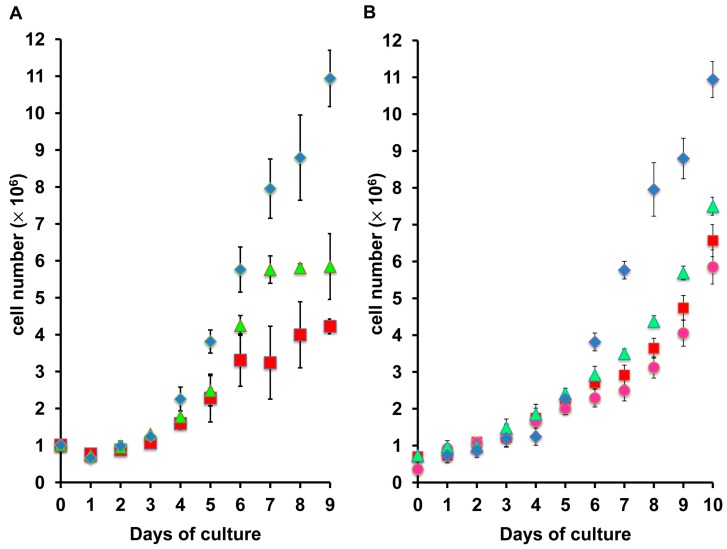
Effect of methionine concentration in culture medium on response of ZFL cells to taurine. Panel (**A**): UltraMEM™-ITES adapted ZFL cells were grown in L-15/FBS (blue diamonds), or UltraMEM™-ITES supplemented with 0 (red squares) or 2 mM taurine (green triangles). Panel (**B**): UltraMEM™-ITES adapted ZFL cells were grown in L-15/FBS (blue diamonds), or UltraMEM™-ITES/500 supplemented with 0 μM (red squares), 12 μM (green triangles), or 160 μM taurine (pink circles). Cells were counted daily. Fifty percent (50%) of each medium was replaced every other day. Data are presented as the mean ± S.D. (*n* = 3 replicates).

Figure 2 showed that even with taurine supplementation, ZFL cells growing in UltraMEM™-ITES had a slower doubling time than L15/FBS. However, an examination of the growth curve showed that at low densities, the cells had a doubling time similar to cells growing in L-15/FBS, but reached saturation density very quickly (Figure 4A). Taurine (2 mM) decreased the doubling time at higher densities and increased the saturation density. However, in comparison, cells growing in L15-FBS continued to grow exponentially over the time course observed, suggesting that both taurine-supplemented and unsupplemented UltraMEM™-ITES media are limiting in some essential nutrient(s). In addition to lacking taurine, UltraMEM™-ITES has a methionine concentration of only 50 μM in contrast to L-15 which has 500 μM Met (Table 2). Furthermore, after only 24 h in culture, the methionine concentration of UltraMEM™-ITES was reduced to approximately 40% of its initial concentration, suggesting that the methionine concentration is limiting (Table 3). Since methionine is the substrate for taurine biosynthesis, it was of interest to investigate the effect of increased methionine concentration on the growth response to taurine in UltraMEM™-ITES adapted cells. The growth of cells in L-15/FBS was compared to that in UltraMEM™-ITES/500 μM methionine with or without taurine supplementation (Figure 4B). With methionine supplementation, saturation density was not reached over the course of the growth curve. Twelve micromolar (12 μM), but not 160 μM taurine, decreases doubling time.

## 3. Discussion

The recent approval of the use of taurine as a feed additive to fish feed by the Food and Drug Administration [47] following the earlier approval by the European Food Safety Authority [48] will increase the need for a wider understanding of taurine homeostasis in fish species of interest to aquaculture. This successful adaptation of ZFL cells to growth in UltraMEM™-ITES has allowed the first definitive investigation of the effects of taurine on expression of taurine biosynthetic pathway and taurine transporter genes in a defined cell type and has shown that taurine supplementation can stimulate growth. With a large excess of taurine in the medium, the intracellular taurine content of ZFL cells in a defined synthetic medium can be increased more than threefold above the basal value provided by biosynthesis. These data indicate that the transport capacity of ZFL cells for exogenous taurine in UltraMEM™-ITES is much higher than their capacity for endogenous taurine biosynthesis. Previous studies have shown that taurine transport capacity can be downregulated by the dietary intake of taurine in mammals [49] and that TauT is important for maintenance of taurine levels in fish [50,51,52,53]. Taurine has also been shown to downregulate TauT transcript levels in cultured rat astrocytes, although not those for taurine biosynthetic enzymes [46]. The effects of taurine concentration have been examined in the culture medium of turbot muscle cells [54]. Although these studies claimed to be in taurine-free L-15 medium, amino acid levels were not measured either in the medium or in the cells. Our studies here have demonstrated that L-15 in fact contains 12 μM taurine. The studies in cultured turbot cells also claimed that the cells were adapted to serum-free conditions. However, the adaptation period used was 24 h, a time presumably sufficient to allow reduction of cellular taurine levels (not shown), but in fact quite inadequate to generate cells accommodated to survive in serum-free conditions. Nevertheless, consistent with our findings, transcript levels for TauT were reduced by 60% at 100 μM taurine, compared presumably to 12 μM.

Although not directly comparable, the ZFL responses are in contrast to what has been reported for zebrafish grown for eight weeks on diets with low (0.02 ± 0.001%) or high (4.08 ± 0.21%) levels of taurine [30]. Whole body taurine levels, expressed as a percentage of body weight, of 1.37 ± 0.03% were found in fish fed the low taurine diet versus 2.04 ± 0.28% in fish fed the high taurine diet. In the liver of fish fed the low taurine diet, transcript levels of ADO and CSAD were significantly higher than in fish fed the low taurine diet, although no differences were seen in the transcript levels of CDO and TauT [41]. This may reflect the observations of Eide et al. who demonstrated a reduced capacity of ZFL cells to upregulate transcription of key genes involved in xenoestrogen responses, compared to primary hepatocytes [55]. Alternatively, it could reflect the differences in the period of exposure or the response of cultured cells that are not under the same metabolic or proliferative control as cells within the animal.

The serum-free adapted cell line described here will allow more in-depth future studies of taurine homeostasis and the biosynthetic capacity of zebrafish cells, such as an investigation of the levels and activities of the biosynthetic enzymes. Certainly, this cell line highlights the importance of monitoring amino acid levels in deciphering the effects/requirements of dietary taurine and will allow a focus on regulation of TauT by sulfur amino acids. Although transcript levels of CDO, CSAD, and ADO do not change in response to taurine supplementation, CDO and CSAD are known to be regulated post-transcriptionally. CDO is one of the most highly regulated metabolic enzymes responding to dietary cysteine. In mammals, it undergoes up to 45-fold changes in concentration and up to 10-fold changes in catalytic efficiency (reviewed in [56]). Cellular CDO concentrations are tightly regulated by the rate of proteasomal degradation as controlled by polyubiquitination [57,58,59]. CDO concentrations can change up to 45-fold [60], multiplied by the potential 10-fold difference in catalytic efficiency [61], for a total potential change in CDO activity of up to 450-fold [40]. CSAD, a pyridoxal-5′-phosphate (PLP)-dependent enzyme, is the rate-limiting enzyme for taurine biosynthesis in mouse [62]. In a CSAD null mouse, the plasma levels of taurine are reduced by >80% and most offspring die shortly after birth [63,64]. Similarly, use of the metabolic modeling program, ZebraGEM, suggests that taurine biosynthesis is controlled by CSAD in zebrafish [65]. Knockdown of CSAD in zebrafish embryos significantly reduces taurine level and results in increased mortality and cardiac abnormalities [40]. CSAD activity can be regulated by phosphorylation by protein kinase C [66].

Fish have varied taurine biosynthesis capability, presumably reflecting differences in the expression levels/activities of the key enzymes and the taurine transporter. The cell line adaptation strategy described here and the synthetic medium used should be applicable to other fish cell lines. For cell lines from aquaculture species with a demonstrated taurine dependence such as cobia (*Rachycentron canadum*) [27,30], yellowtail (*Seriola quinqueradiata*) [19,33], seabream (*Pagrus major*) [67], or sablefish [68], it should be possible to adjust cells to the synthetic medium by inclusion of taurine. Taurine could be subsequently withheld to examine effects on the biosynthetic pathways. Such cell lines would be invaluable in elucidating taurine biosynthetic capacity of different species, the relationship to sulfur amino acids, and thus in designing adequate diets for aquaculture species.

## 4. Materials and Methods

### 4.1. Cell Lines and Cell Culture

The ZFL cell line (ATCC #CRL-2643), derived from a pool of 10 adult zebrafish livers, has characteristics of liver parenchymal cells and exhibits characteristics consistent with differentiated liver cell function [69]. ZFL cells have been used to investigate toxicological [70,71], pharmacological [72], and innate immune function studies in fish [73]. Cells were maintained in Leibovitz’s L-15 medium (Cellgro, Manassas, VA, USA), supplemented with 20 mM HEPES-KOH, pH 7, 2 mM sodium pyruvate, 100 units·mL^−1^ of penicillin and streptomycin, and supplemented with 9% fetal bovine serum (Atlanta Biologicals, Lawrenceville, GA, USA) (L15-FBS) in 100 mm tissue culture plates at 28 °C, without sodium bicarbonate and CO_2_ ([74]).

UltraMEM™-ITES (Lonza, Walkersville, MD, USA) is a protein-free basal medium supplemented with insulin, transferrin, ethanolamine, selenium (ITES) and l-glutamine. Recombinant human insulin and transferrin, both at 20 μg·mL^−1^, are the only protein components. ZFL cells maintained in L15-FBS were initially transferred into medium containing 10% UltraMEM™-ITES with 20 mM HEPES-KOH, pH 7, and 2 mM sodium pyruvate. After two passages, cells were transferred to L15-FBS containing 20% UltraMEM™-ITES. Over 123 days in culture, L15-FBS was exchanged with increasing concentrations of UltraMEM™-ITES in 10% increments every second passage, going from 0 to 100% UltraMEM™-ITES. Over this period, FBS concentrations decreased concomitantly from 9 to 0%. Cell health was monitored by regular microscopic observation for normal growth.

Cells were counted every 2–8 days by trypsinization in 0.05% trypsin/0.03% ethylenediaminetetraacetic acid (EDTA) in phosphate buffered saline (PBS) for 10 min at room temperature. Average cell counts were calculated from the number of cells in four quadrants. Doubling times (DT) were calculated using the formula DT = T ln2/ln(Xe/Xb), in which T is the incubation time, Xb is the cell number at the beginning, and Xe is the cell number at the end of the period.

### 4.2. Measurement of Medium Amino Acid Concentrations and Cellular Amino Acid Pools

Amino acid levels in culture medium: ZFL cells were cultured in 100 mm plates with 10 mL UltraMEM™-ITES supplemented with 0, 12, 160 μM, or 2 mM taurine for 24 h. At each taurine concentration, the growth medium from three replicate plates was removed and a 1-mL aliquot was taken for amino acid analysis.

Amino acid levels in ZFL cells: cells were washed rapidly two times with 10 mL of precooled (4 °C) PBS. Five milliliters of MeOH, prechilled on dry ice, was added to each plate to disrupt the cells. Cells and liquid were scraped into 15-mL conical tubes. Next, 2 × 4 mL MeOH was used to wash the plates for collection of residual cells and combined with the first cell suspension. The samples were held in liquid nitrogen for 10 min, thawed in an ice bath for 10 min, and briefly vortexed. This freeze–thaw cycle was repeated three times to complete cell disruption. The sample was centrifuged at 4 °C and 3000× *g* for 30 min to remove cell debris and the supernatant was transferred to a new tube. Methanol was removed by drying at 60 °C prior to amino acid analysis. The residue was solubilized in 1 mL of PBS and the protein concentration was quantitated using a Qubit™ Protein Assay kit (Life Technologies Corporation, Eugene, OR, USA) according to the manufacturer’s instructions. After sample normalization based on total protein levels, absolute levels of amino acids in the supernatants were quantified by LS-MC using the Waters AccQ Tag™ method on an Agilent 1200 Infinity Series HPLC with Quaternary Pump and FLD Detector. Statistical analysis of free amino acid pools in media and cells were performed using one-way ANOVA and Tukey’s multiple-range test. A *p*-value of less than 0.05 was taken to indicate statistical significance. Norleucine (50 pmol) was injected with the sample to assess amino acid recovery. The amino acid concentration in the medium is expressed as μM. The level of amino acids in the cells is given as pmol per 3 × 10^6^ cells.

### 4.3. Measurement of Transcript Levels of the Enzymes of Taurine Biosynthesis

RNA was prepared from ZFL cells using the Ambion PureLink total RNA extraction mini kit (Life Technologies Corporation, Carlsbad, CA, USA) following the manufacturer’s protocol. RNA concentration, purity, and integrity were determined by Nanodrop ND-1000 spectrophotometry (NanoDrop Technologies, Wilmington, DE, USA) and automated electrophoresis using the Agilent 2100 BioAnalyzer (Agilent, Santa Clara, CA, USA). Values of >2 for 260/280 and 260/230 ratios were considered to be of sufficient purity. Generation of cDNA amplicons of the predicted size was confirmed by end-point RT-PCR. cDNA was synthesized using Revertaid™ M-MULV reverse transcriptase (Fermentas GmbH, St. Leon-Roth, Germany) and random hexamer primers (Qiagen, Valencia, CA, USA). Amplification of cDNA for large ribosomal protein L13A was used as an internal control. Quantitative PCR (qPCR) was performed on a 7500 Real Time PCR System (Applied Biosystems, Foster City, CA, USA) with SYBR green fluorescence. Data generated were analyzed using 7500/7600 Sequence Detection Software (Life Technologies, Foster City, CA, USA).

Primers used to amplify cDNAs for zebrafish cysteamine dioxygenase (ADO), cysteine dioxygenase (CDO), cysteinesulfinate decarboxylase (CSAD), and taurine transporter (TauT) were designed to span exon-exon junctions based on published zebrafish sequences in GenBank by Primer 3 software (Table 5). The efficiency of the primers (>90% for all pairs) was determined using five different dilutions of cDNA (20, 10, 5, 2.5, and 1.25 ng cDNA per reaction) made from ZFL cell RNA. Primers for ribosomal protein, L13A, used as the reference gene, were based on published primer sequences [75]. Paired *t*-tests (*p* = 0.05) were used to assess the differences in relative expression of the target genes.

## Figures and Tables

**Table 1:** Taurine Concentrations in L-15, FBS, and UltraMEM™-ITES.

Source	Taurine Concentration (Mean ± S.D.)
L-15	11 ± 0.8 μM
FBS	20 ± 4 μM
L-15+FBS	12 ± 1 μM
UltraMEM™-ITES	Not detectable

Amino acid concentrations of L-15, FBS, and UltraMEM™-ITES: Taurine concentration was measured by liquid chromatography/mass spectrometry as described in Section 4.2 [41]. FBS, fetal bovine serum.

**Table 2:** Amino acid concentrations (μM) of UltraMEM™-ITES before and after incubation with ZFL cells for 24 h.

	At 0 h	After 24 h			
Amino Acid	0 μM	+0 μM	+12 μM	+160 μM	+2 mM
B (D/N)	68	63 ± 1	64 ± 2	58 ± 4	75 ± 5
S	939	774 ± 19	778 ± 27	682 ± 65	817 ± 56
Z (E/Q)	68	91 ± 3	94 ± 1	88 ± 7	120 ± 8
G	85	140 ± 7	143 ± 2	127 ± 9	152 ± 10
H	774	851 ± 32	863 ± 17	743 ± 69	836 ± 49
HYPOTAURINE	0.00	2 ± 0.1	3 ± 0.02	0.0 ± 0.0	0.0 ± 0.0
TAURINE	0.00	0.00 ± 0.0	7.4 ± 0.2	112 ± 10	1,963 ± 131
R	263	245 ± 9	244 ± 6	219 ± 22	236 ± 19
T	249	160 ± 6	158 ± 7	141 ± 13	170 ± 12
A	105	662 ± 5	702 ± 7	613 ± 56	789 ± 37
P	35	25 ± 4	19 ± 1	17 ± 1	21 ± 3
Y	78	73 ± 2	72 ± 1	65 ± 5	76 ± 4
V	462	404 ± 14	407 ± 9	358 ± 28	429 ± 28
METHIONINE	46	19 ± 2	16 ± 2	15 ± 1	14 ± 1
K	170	156 ± 5	158 ± 4	140 ± 10	165 ± 11
I	210	126 ± 4	123 ± 3	110 ± 8	130 ± 9
L	595	471 ± 17	473 ± 2	416 ± 32	506 ± 32
F	68	35 ± 3	32 ± 2	30 ± 3	28 ± 2
	Recovery of norleucine (pmol per sample with 50 pmol load)				
NORLEUCINE *		50 ± 2	50 ± 2	51 ± 1	51 ± 5

Amino acid concentrations (μM) in UltraMEM™-ITES before and after 24 h incubation with ZFL cells. ZFL cells, fully adapted to growth in 100% UltraMEM™-ITES, were incubated in 0 μM, 12 μM, 160 μM, or 2 mM taurine for 24 h. Amino acid concentrations were measured by LC-MS, as described in Section 4.2 [41]. The amino acid analysis method used converts glutamine and asparagine to their respective acids so values presented represent both amino acid forms. * Norleucine (50 pmol) was injected with each sample to assess amino acid recovery; the value is calculated from the fluorescence area of the norleucine peak injected. Samples were normalized based on norleucine recovery and represent the mean of triplicates ± standard deviation.

**Table 3:** Amino acid levels in ZFL cells growing in UltraMEM™-ITES with/without taurine supplementation (pmol per 3 × 10^6^ cells).

	Medium at 0 h (μM)	Cellular Concentration after 24 h (pmol per 3 × 10^6^ Cells)			
Amino Acid		+0 μM	+12 μM	+160 μM	+2 mM
B (D/N)	68	73 ± 16	83 ± 6	87 ± 13	115 ± 6
S	939	238 ± 9	236 ± 11	223 ± 27	254 ± 16
Z (E/Q)	68	593 ± 22	732 ± 40	720 ± 100	654 ± 33
G	85	94 ± 21	119 ± 3	112 ± 11	135 ± 13
H	774	37 ± 13	33 ± 4	31 ± 7	32 ± 1
HYPOTAURINE	0.00	41 ± 10	61 ± 3	61 ± 12	62 ± 6
TAURINE	0.00	315 ± 63	566 ± 34	862 ± 34	1008 ± 44
R	263	18 ± 5	ND	ND	ND
T	249	54 ± 8	59 ± 5	57 ± 10	66 ± 4
A	105	307 ± 59	348 ± 26	340 ± 41	424 ± 21
P	35	53 ± 8	55 ± 10	51 ± 10	62 ± 2
Y	78	19 ± 2	20 ± 2	20 ± 3	22 ± 1
V	462	86 ± 17	93 ± 9	91 ± 10	103 ± 4
METHIONINE	46	9 ± 1	8 ± 0.2	8 ± 0.2	8 ± 0.20
K	170	21 ± 3	19 ± 1	20 ± 0.6	22 ± 0.1
I	210	33 ± 5	34 ± 5	34 ± 3	37 ± 0.3
L	595	94 ± 23	105 ± 9	100 ± 9	118 ± 6
F	68	24 ± 5	25 ± 4	23 ± 2	23 ± 2
	Recovery of norleucine (pmol per sample with 50 pmol load)				
NORLEUCINE *		54 ± 3	54 ± 3	53 ± 2	53 ± 2

Effect of taurine supplementation on amino acid levels in ZFL cells grown in UltraMEM™-ITES. ZFL cells, fully adapted to growth in 100% UltraMEM™-ITES, were exposed to 0 μM, 12 μM, 160 μM, and 2 mM taurine for 24 h. Triplicate plates, each with an average of 3 × 10^7^ cells, were used for each condition. Protein recovered from each condition was 29 ± 2, 28 ± 0.7, 25 ± 0.9, and 22 ± 1.5 pg/cell protein, respectively. * Norleucine (50 pmol) was injected with each sample to assess amino acid recovery; the value is calculated from the fluorescence area of the norleucine peak injected. Samples were normalized based on total protein levels and norleucine recovery and represent the mean of triplicates ± standard deviation. Amino acid levels were measured as described in the legend for Table 2 and are expressed as pmol per 3 × 10^6^ cells.

**Table 4:** Fold change in intracellular levels of taurine, hypotaurine, and methionine in response to taurine supplementation of the medium.

Taurine Supplementation	Fold Change in Intracellular Taurine Level	Fold Change in Intracellular Hypotaurine Level	Fold Change in Intracellular Methionine Level
12 μM	1.8	1.5	0.9
160 μM	2.8	1.5	0.9
2 mM	3.2	1.5	0.9

**Table 5:** Primer pairs used for quantitative reverse transcription PCR (RT-qPCR).

Gene	Forward Primer	Reverse Primer	GenBank Accession Number
*D. rerio* ADO	5′-TTACAGACTGCTGGGAAAAA-3′	5′-GGCTTGAAACAAGCAAATAA-3′	NM_001008634.1
*D. rerio* CDO	5′-GAACCTGATGGAGTCCTACC-3′	5′-AACTTTCCGTTTCCTTCATC-3′	NM_200741.1
*D. rerio* CSD	5′-AGCTGAGATCTCTCCTGGAC-3′	5′-TGGTATTGAGGGTTTCAGTG-3′	NM_001007348
*D. rerio* TauT	5′-ATCACCTGTTGGGAGAAACT-3′	5′-CAGGTAGTACAAGCCACAGG-3′	NM_001037661.1
*D. rerio* L13A	5′-TCTGGACTGTAAGAGGTATGC-3′	5′-AGACGCACAATCTTGAGAGCAG-3′	NM_212784.1

Primer pairs used for RT-qPCR. Sequences of the primer pairs used for RT-qPCR determination of the transcript levels of zebrafish cysteamine dioxygenase (ADO), cysteine dioxygenase (CDO), cysteinesulfinate decarboxylase (CSAD), taurine transporter (TauT), and ribosomal protein L13A (L13A).
